# Genome Reduction and Secondary Metabolism of the Marine Sponge-Associated Cyanobacterium *Leptothoe*

**DOI:** 10.3390/md19060298

**Published:** 2021-05-24

**Authors:** Despoina Konstantinou, Rafael V. Popin, David P. Fewer, Kaarina Sivonen, Spyros Gkelis

**Affiliations:** 1 Department of Botany, School of Biology, Aristotle University of Thessaloniki, GR-541 24 Thessaloniki, Greece; kidespoi@bio.auth.gr; 2 Department of Microbiology, University of Helsinki, Viikinkaari 9, FI-00014 Helsinki, Finland; rafael.popin@helsinki.fi (R.V.P.); david.fewer@helsinki.fi (D.P.F.); kaarina.sivonen@helsinki.fi (K.S.)

**Keywords:** cyanobacteria, symbionts, marine sponges, comparative genomics, natural products, biosynthetic gene clusters

## Abstract

Sponges form symbiotic relationships with diverse and abundant microbial communities. Cyanobacteria are among the most important members of the microbial communities that are associated with sponges. Here, we performed a genus-wide comparative genomic analysis of the newly described marine benthic cyanobacterial genus *Leptothoe* (Synechococcales). We obtained draft genomes from *Le. kymatousa* TAU-MAC 1615 and *Le. spongobia* TAU-MAC 1115, isolated from marine sponges. We identified five additional *Leptothoe* genomes, host-associated or free-living, using a phylogenomic approach, and the comparison of all genomes showed that the sponge-associated strains display features of a symbiotic lifestyle. *Le. kymatousa* and *Le. spongobia* have undergone genome reduction; they harbored considerably fewer genes encoding for (i) cofactors, vitamins, prosthetic groups, pigments, proteins, and amino acid biosynthesis; (ii) DNA repair; (iii) antioxidant enzymes; and (iv) biosynthesis of capsular and extracellular polysaccharides. They have also lost several genes related to chemotaxis and motility. Eukaryotic-like proteins, such as ankyrin repeats, playing important roles in sponge-symbiont interactions, were identified in sponge-associated *Leptothoe* genomes. The sponge-associated *Leptothoe* stains harbored biosynthetic gene clusters encoding novel natural products despite genome reduction. Comparisons of the biosynthetic capacities of *Leptothoe* with chemically rich cyanobacteria revealed that *Leptothoe* is another promising marine cyanobacterium for the biosynthesis of novel natural products.

## 1. Introduction

Sponges host abundant and remarkable diverse microbial communities [1] that exhibit biological complexity similar to the human microbiome [2,3]. Cyanobacteria are an ancient lineage of photosynthetic prokaryotes demonstrating ecological key roles (e.g., oxygen production, nitrogen fixation, carbon flux) in a broad range of habitats, including sponges [4,5], with which they are often found in symbiosis (cyanobionts). Several approaches including whole-genome sequencing of symbiotic microbes and metagenomic binning have provided insights into the functional potential of the symbionts [6,7]. For instance, it has been shown that across sponge-associated bacteria taxa there are pathways involved in carbon fixation, B-vitamin synthesis, taurine metabolism, sulfite oxidation, and most steps of nitrogen metabolism [7,8]. *Candidatus Synechococcus spongiarum,* a widespread (yet uncultivated) sponge symbiont, has specific adaptations to life inside sponges [9]. This obligate cyanobiont showed extreme genome reduction [9], similarly to other bacterial sponge symbionts such as *Candidatus Endohaliclona renieramycinifaciens* [10]. Genome reduction is the major genomic feature of bacterial symbionts [11] and is thought to be a process that reduces the cost of genome replication [12].

Cyanobacteria are a prolific source of natural products with complex chemical structures and interesting bioactivities [13]. Advances in genomics have greatly expanded our knowledge and understanding of cyanobacterial natural product biosynthesis [13] with known natural products linked to biosynthetic gene clusters and new natural products discovered through genome mining (e.g., [14,15,16,17]). However, the majority of natural products from cyanobacteria are described from a relative limited number of genera [13]. This phenomenon is attributed to problems with cyanobacterial taxonomy that obfuscates the true distribution of natural products in marine cyanobacteria.

Marine sponges are also prolific sources of natural products of great interest for drug development, contributing to nearly 30% (more than 4850 compounds) of all marine natural products discovered [18]. Sponge-associated bacteria are widely thought to be responsible for sponge natural product diversity [2,3,19], with cyanobacteria being among the major producers [20].

Sponge-associated members of the newly described marine cyanobacterium *Leptothoe* [21,22] were found to be highly cytotoxic against human breast, skin, and colon cancer epithelial cells [23]. Extracts of *Le. kymatousa* TAU-MAC 1615 were found to have antibacterial activity [23]. In this study, we sequenced two draft *Leptothoe* genomes, *Le. kymatousa* TAU-MAC 1615 and *Le. spongobia* TAU-MAC 1115, previously isolated form the marine sponges *Chondrilla nucula* and *Acanthella acuta*, respectively [21,24]. We performed comparative genomic analyses of sponge-associated, other host-associated and free-living members of *Leptothoe* genus, and other marine cyanobacterial genera, aiming to (a) identify symbiosis factors and (b) gain insight into their natural product biosynthetic potential.

## 2. Results and Discussion

Two new sponge-associated cyanobacterial draft genomes belonging to the newly described *Leptothoe* genus were recovered from the isolates TAU-MAC 1615 and 1115 (Figure S1, Table 1). To date, most microbial isolates derived from sponges have been mainly affiliated with Proteobacteria [7,25,26]. A limited number of cyanobacterial strains have been isolated from sponges [24]. Indeed, only one genome assembly from a sponge-associated cyanobacterial strain is available in public databases for a *Myxosarcina*-like cyanobacterium isolated from *Terpios hoshinota* [27]. Recently, a considerable number of novel genera and species of cyanobacteria were found to be associated with Aegean Sea sponges [21,22].

**Figure 1:**
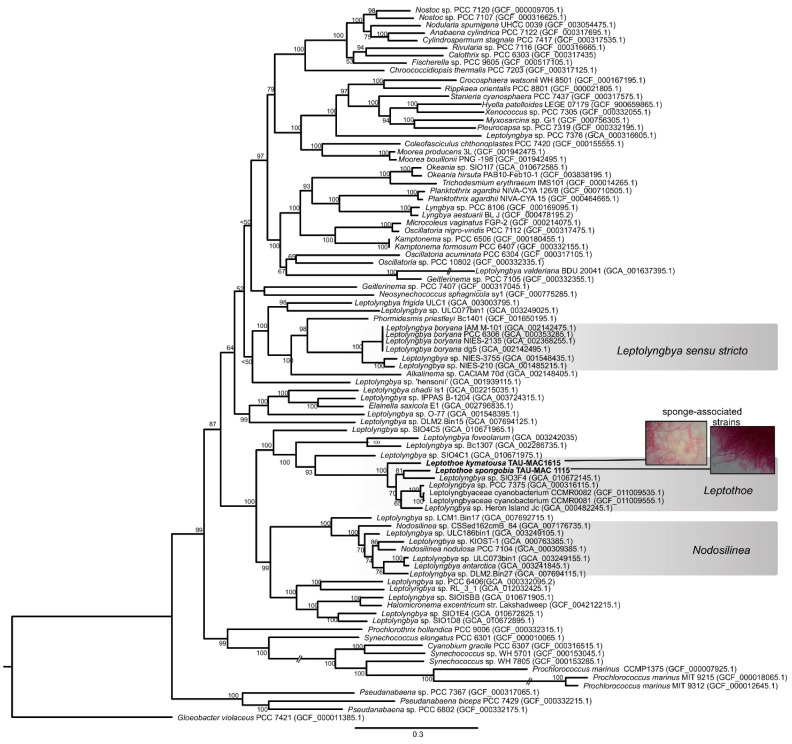
Maximum likelihood phylogenomic tree based on 120 conserved proteins in cyanobacterial genomes. The sponge-associated *Leptothoe* strains sequenced in the present study are shown in bold. Accession numbers of the sequences are presented in parentheses.

The size of the assembled sponge-associated *Leptothoe* genomes ranged between 4.06 Mb for *Le. kymatousa* TAU-MAC 1615 and 5.24 Mb for *Le. spongobia* TAU-MAC 1115, with G+C contents of 50.5% and 47.3%, respectively (Figure S1, Table 1). Table 1 summarizes the basic genome features of sponge-associated *Leptothoe* genomes, *Leptothoe* genomes associated with other macro-organisms (isolated from turfs), and free-living *Leptothoe* genomes used for comparative genomic analyses in this study after verification of genome-wide relatedness using phylogenomic approaches (Figure 1). The draft assemblies of sponge-associated strains showed the smallest genome sizes (Table 1). Although the genome coverage of sponge-associated strains was high (approximately ×450 and ×90 for TAU-MAC 1615 and TAU-MAC 1115, respectively), the genome completeness estimated by the marker sets used by CheckM (encode essential functions) was substantially complete (≈70%) (Table 1). The GC% contents of the strains were quite similar, varying from 47.3% (TAU-MAC 1115, CCMR0081) to 50.5% (TAU-MAC 1615). Concerning the number of scaffolds, the SIO3F4 metagenome showed the highest number (1508) and the lowest N50 (7412). The number of coding sequences (CDSs) of sponge-associated strains was considerably lower (3638–4790) than the rest of the *Leptothoe* strains, while the amount of CDSs categorized in RAST subsystems (13–17%) was quite similar (Table 1). The remaining 84–87% of CDSs were not classified in any subsystem.

The phylogenomic reconstruction based on 120 bacterial single-copy conserved marker genes indicated that our two sponge-associated strains were placed inside *Leptothoe* clade and grouped together with 5 of the 35 public cyanobacterial genomes identified as *Leptolyngbya* or Leptolyngbyaceae (Figure 1). *Leptothoe* is a thin marine filamentous cyanobacterium with benthic lifestyle being epilithic, epizoic, or epiphytic, recently separated from the polyphyletic filamentous genus *Leptolynbya* by a taxonomic re-evaluation [21]. 

The remaining 30 cyanobacteria, identified as *Leptolyngbya* or Leptolyngbyaceae, were grouped in different clades spread across a variety of cyanobacteria orders (Figure 1). This analysis further supported the evolutionary divergence of *Leptothoe* from *Leptolyngbya* sensu stricto and other thin filamentous genera separated from *Leptolyngbya* such as *Nodosilinea* and *Elainella* (Figure 1). The analysis also supported the evolutionary distinction of the two sponge-associated strains, TAU-MAC 1115 and TAU-MAC 1615, which belong to different species *Le. spongobia* and *Le. kymatousa* (Figure 1). The free-living strains PCC 7375 and Heron Island J were placed separately within a subclade along with two strains (CCMR0081 and CCMR0081) isolated from turf samples growing over corals, while the metagenome-assembled genome SIO3F4 isolated from turfs assembles in Panama was placed in the same subclade with *Le. spongobia* TAU-MAC 1115 (Figure 1). Further, our phylogenomic analysis showed that the members of genus *Leptothoe* (seven in total) were not grouped on the basis of the isolation source (host-associated or free-living), indicating the lack of host-specific clustering. Other marine bacterial genera, including free-living and host-associated members such as *Pseudovibrio*, have shown a lack of host-specific clustering [30]. However, the sponge-associated *Le. kymatousa* TAU-MAC 1615 was placed separately in the phylogenomic tree, likely suggesting its independent evolution from other host-associated strains of the genus. These results might indicate distinct patterns of evolution among members of genus *Leptothoe*. 

### 2.1. Genomic Hallmarks of a Symbiotic Lifestyle

#### 2.1.1. Genome Reduction of Sponge-Associate Strains

**Figure 2:**
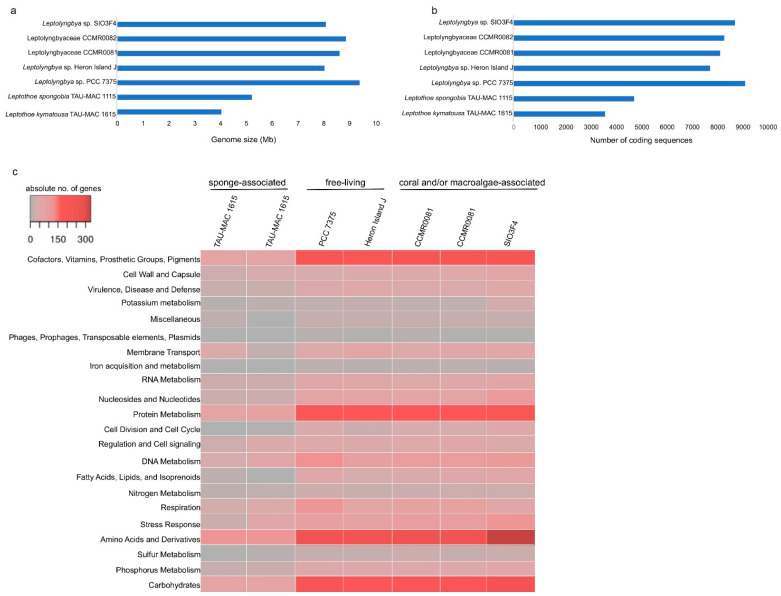
Comparison of genome size (**a**), number of coding sequences (**b**), and number of genes in each RAST subsystem [28] (**c**) of the analyzed *Leptothoe* genomes.

*Leptothoe kymatousa* TAU-MAC 1615 and *Leptothoe spongobia* TAU-MAC 1115 are not obligate sponge symbionts and can sustain growth in pure cultures. However, these two strains showed considerable smaller genome size (Figure 2a). This pattern of genome reduction was also observed for the obligate sponge cyanobiont *Candidatus Synechococcus spongiarum* [9,31]. Genome reduction is widely observed mainly in obligate bacterial symbionts [11], although signs of genome reduction have been identified in facultative symbionts too [32]. Both cyanobionts are slow growing in culture conditions, and thus they could be considered as facultative symbionts that are generally not essential for the host’s survival, although they may contribute to host fitness. Recently evolved symbionts, which have undergone genome reduction, are generally characterized by a proliferation of pseudogenes and mobile elements [11]. The two *Leptothoe* sponge-associated genomes reported here showed lower numbers of those traits compared to the other members of the genus (Table 1); on the basis of these data, we hypothesized that sponges did not recently acquire the *Leptothoe* symbionts. 

The number of coding sequences of the sponge-associated strains were also half compared to the remaining strains (Figure 2b). An overview of different subsystems obtained using the RAST and SEED annotation showed that the sponge-associated *Leptothoe* strains harbored considerably fewer genes related to several essential functions (Figure 2c, Table S1). Remarkably, *Le. kymatousa* TAU-MAC 1615 and *Le. spongobia* TAU-MAC 1115 have undergone extreme reduction of the number of genes encoding for cofactors, vitamins, prosthetic groups, pigments, proteins, and amino acid biosynthesis (Figure 2c, Table S1). Sponge-associated strains may be dependent on co-occurring microbes for lost metabolic capacities [33]. A complete loss of genes involved in DNA recombination and fewer genes involved in DNA repair were observed in their genomes, while they retained approximately the same number of genes for DNA replication as the rest of the *Leptothoe* genomes. The sponge-associated strains have lost several genes involved in potassium, sulfur, and phosphorus metabolism (Table S1). They were also found to have fewer genes related to stress response, mainly genes coding for antioxidant enzymes, as well as fewer genes responsible for the biosynthesis of capsular polysaccharide (CPS) and extracellular polysaccharides (Table S1). Our *Leptothoe* genomes were characterized by a complete or near complete lack of chemotaxis and motility traits, which are among the most depleted functions in sponge-associated bacteria genomes [34]. 

**Figure 3:**
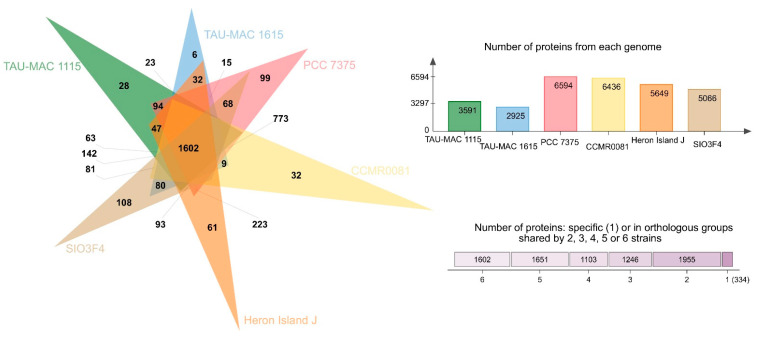
Analysis of homologous protein clusters in the genomes of *Le. kymatousa* TAU-MAC 1615, *Le. spongobia* TAU-MAC 1115, Leptolyngbyaceae sp. CCMR0081, Leptolyngbyaceae sp. CCMR0082, *Leptolyngbya* sp. SIO3F4 *Leptolyngbya* sp. PCC 7375, and *Leptolyngbya* sp. Hero Island J.

The number of COGs (clusters of orthologous groups of proteins) per genome ranged from 6594 in free-living PCC 7375 strain to 2925 in the sponge-associated TAU-MAC 1615 strain. *Leptothoe* strains shared > 80% average amino acid identity (AAI) (Figure S2), while only 1602 COG entries that account for approximately half (for sponge-associated strains) or even lower (≈25% for the rest of the strains) of the total number of COG entries were common in all genomes (Figure 3), likely suggesting specific adaptations for different lifestyles and for different symbiont types. Genome streamlining process forces adaptations of cyanobacterial genomes to specific niches that are also reflected in their different functional capacities [12]. Previously, sponge-associated and free-living *Synechococcus* genomes have also been found to share half of their total number of COGs, suggesting variability and specific adaptations of each member of the genus [9]. Comparisons based on COG categories among the sponge-associated, coral, and/or macroalgae-associated and free-living *Leptothoe* revealed a relative lower abundance of genes belonging to the different functional categories in the sponge-associated strains. This analysis identified an overrepresentation of functional categories ‘J’ (translation, ribosomal structure, and biogenesis), ‘L’ (replication, recombination, and repair), ‘O’ (posttranslational modification, protein turnover, chaperones), and ‘P’ (inorganic ion transport and metabolism) and was observed in the genomes of the coral and/or macroalgae-associated strains (Figure S3). A uniform distribution of genes belonging to COG functional categories between the two sponge-associated strains was detected.

**Figure 4:**
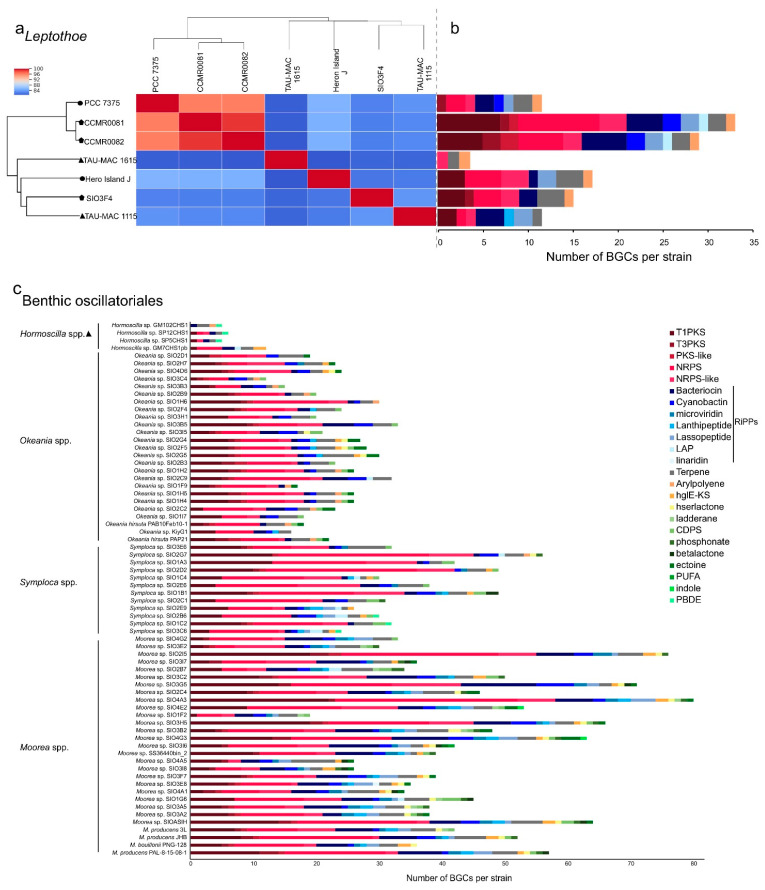
Average nucleotide identity heatmap (**a**) and composition of BGCs identified in *Leptothoe* genomes (**b**) and composition of BGCs identified in marine benthic filamentous Oscillatoriales (**c**). The absolute number of BGCs per strain assigned to each BGC class is shown. Triangle, sponge associated strains; polygon, coral and/or macroalgae-associated strains; circle, free-living strains.

The sponge-associated strains possess biosynthetic gene clusters (BGCs) encoding for natural products despite undergoing genome reduction (Figure 4a,b). It has been proposed that maintenance of such clusters sustains the symbiotic interaction [35]. Natural product BGCs were previously detected in another cyanobacterial sponge symbiont, *Hormoscilla spongilae,* suggesting that these biosynthetic capacities to produce metabolically expensive natural products may contribute to host fitness [36].

#### 2.1.2. Eukaryotic-Like Protein (ELP)-Encoding Genes

Genes encoding eukaryotic-like proteins (ELPs) such as ankyrin-like domains (ANKs), tetratricopeptide repeats (TPRs), Leucine-rich repeat (LRR) protein, and pyrroloquinoline quinone (PQQ) were detected in the two sponge-associated *Leptothoe* strains (Table 2); ELPs are often detected in facultative or obligate symbionts and play a key role in the modulation of cellular protein–protein interactions [37,38]. In particular, abundance of ANKs seems to be a major genomic feature of sponge symbionts [6,26] as they have been thought to be involved in preventing phagocytosis by the sponge host. Indeed, the role of ANKs in modulating the amoebal phagocytosis in sponge symbionts was experimentally validated [39]. Genome analyses of sponge-associated Alphaproteobacteria [7,26,30], Deltaproteobacteria [40], and Poribacteria [41] have revealed the presence of ELPs, as well as the particular abundance of ANKs. The obligate sponge symbionts *Hormoscilla spongeliae* and *Candidatus Synechococcus spongiarum* had a great number of ELP repeats, while different free-living cyanobacteria taxa such as *Nodosilinea*, *Leptolyngbya*, *Synechococcus*, *Prochlorococcus*, and *Cyanobium gracile* almost lacked ANK domains, the typical genomic signature of sponge symbionts (Table 2). We also detected the different ELP types in varying proportions in other host-associated and free-living members of the genus *Leptothoe*. Similarly, ELPs have been previously detected in host-associated and free-living members of Alphaproteobacteria, likely suggesting the ability of strains to infect a different range of marine hosts and attach to various marine niches [26,30].

### 2.2. Biosynthetic Potential of Leptothoe

The genomic repertoire for secondary metabolism of seven *Leptothoe* genomes was predicted using antiSMASH (Figure 4a,b). *Leptothoe* genomes were found to harbor a considerable number of BGCs (121), the majority of which have no known end product. BGCs with unknown end products are present in almost all cyanobacterial genomes [42], and on the other hand, there are still natural products for which a biosynthetic origin is unknown [43]. The vast majority of BGCs in *Leptothoe* genomes were predicted to encode non-ribosomal peptide synthetases (NRPS) (24 BGCs), followed by type I polyketide synthase (T1PKS) (20 BGCs) and bacteriocin (17 BGCs) (Figure 4, Table S2). Previously, genome-mining efforts have revealed that a major fraction of cyanobacterial natural products is produced using NRPS or PKS enzymes systems [43,44]. Bacteriocin BGCs, which are widespread in cyanobacterial genomes [45], were detected in almost all *Leptothoe* genomes (except for *Le. kymatousa* TAU-MAC 1615). Bacteriocins have been mainly reported to exhibit antimicrobial activity [46], but are also promising as antivirals, plant protection agents, and anticancer agents [47]. Further, it is suggested that bacteriocins may be involved in shaping bacterial communities through inter- and intra-specific interactions [47]. In addition, lassopeptide and terpene synthase BGCs were detected with high relative abundance in almost all the *Leptothoe* genomes, while cyanobactin and arylpolyene BGCs were rarely found in some of the genomes. Terpene BGCs, reported in a wide variety of bacteria including cyanobacteria [48], were also present in all *Leptothoe* genomes. The considerably similar *Leptothoe* strains CCMR0081 and CCMR0081 (96% average nucleotide identity) associated with corals and macroalgae (isolated from turfs) showed the highest number of natural product BGCs (Figure 4a,b, Table S2). In contrast, the two sponge-associated *Leptothoe* species (sharing ≈84% average nucleotide identity) showed the lowest number of natural product BGCs. Interestingly *Le. spongobia* strain harbored a BGC encoding for a lanthipeptide (Figure 4b). Lanthipeptides are ribosomally synthesized and post-translationally modified peptides (RiPPs) that display a wide variety of biological activities [49], while their detection and isolation are restricted to bacteria [43]. Lanthipeptide BGCs are particularly found in the genomes of many genera of Firmicutes, Actinobacteria, Proteobacteria, Bacteroidetes, and Cyanobacteria [50].

**Figure 5:**
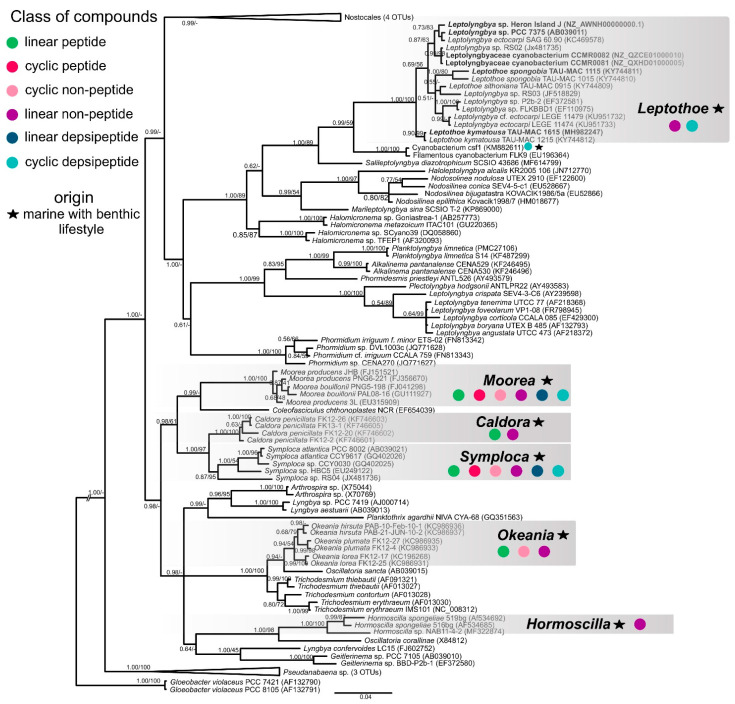
Phylogenetic relationships of *Leptothoe* strains based on the 16S rRNA gene sequence, in relationship to representative strains of other marine filamentous cyanobacteria with benthic lifestyle, with *Gloeobacter* violaceus as outgroup. The tree was constructed with the Bayesian inference (ΒΙ) method and the maximum-likelihood (ML) method; BI topology is demonstrated. Support values are indicated as posterior probability for Bayesian inference and bootstrap support for maximum likelihood analysis. The bar represents 0.04 nucleotide substitutions per site.

We assigned producers of known natural products to the *Leptothoe* lineage by combining 16S rRNA phylogenetic analysis with the “Comprehensive database of secondary metabolites from cyanobacteria ‘CyanoMetDB’” [51]. We searched the database for entries attributed to *Leptolyngbya*, *Pseudanabaena* (*Pseudanabaena persicina* = *Leptolyngbya ectocarpi*), *Phormidium* (*Phormidium ectocarpi* = *Leptolyngbya ectocarpi*), or thin filamentous strains of pinkish color, and where a sequence was available, it was included in our phylogenetic analysis (Table S3, Figure 5). This analysis demonstrated that two strains previously assigned to *Leptolyngbya*, *Leptolyngbya ectocarpi* SAG 60.90, and *Leptolyngbya* sp. RS03, reported to produce compounds such as hierridin B, grassypeptolides D and E, a lyngbyastatin analogue, and dolastatin 12, belong to the *Leptothoe* genus (Table S3, Figure 5). However, the natural product BGCs involved in the biosynthesis of the abovementioned compounds have not been studied as the genomes of these *Leptothoe* strains have not been sequenced yet. Our phylogenetic analysis revealed that *Leptothoe* was closely affiliated with three other marine benthic cyanobacteria, *Salileptolyngbya* and two strains with unknown taxonomic status (Cyanobacterium csf1 and Filamentous cyanobacterium FLK9); csf1 was found to produce two new cyclic depsipeptides, companeramides A and B (Figure 5; CyanoMetDB). Further, in our analysis, other genera of marine origin with benthic lifestyle and often with a reddish to pinkish thallus color, known for the production of natural products such as linear and cyclic peptides, linear and cyclic non-peptides, and linear and cyclic depsipeptides, were extracted from the CyanoMetDB database. These chemically rich genera—*Moorea*, *Caldora*, *Symploca*, *Okeania*, and *Hormoscilla*—were placed in separate clades inside Oscillatoriales and were found to be distantly related to *Leptothoe* (Figure 5).

Most of the natural products from marine cyanobacteria have been isolated from *Moorea* [52], which occur in high densities in marine environments of tropical and sub-tropical regions, making the harvest of biomass easily accessible [52]. Similarly, *Symploca*, *Caldora,* and *Okeania* form large populations attached to hard substrates in marine habitats [53,54] and yield a great number of natural products. Novel compounds with strong anticancer properties such as apratoxins, grassypeptolides, wewakazole B, odoamide, and caldoramide are isolated from these marine genera [55,56,57,58,59]. Interestingly, cyclic depsipeptides are the main peptides with cytotoxic effects isolated from marine cyanobacteria, including 76 compounds [60]. Genome-mining analysis conducted in the present study for >70 marine cyanobacteria also highlighted the high metabolic potential of the well-studied Oscillatoriales; numerous BGCs were identified in their genomes (Figure 4c). On the other hand, other marine filamentous cyanobacteria with smaller trichomes and a slower growth rate, such as *Leptolyngbya*-like or *Pseudanabaena*-like, have been overlooked [52], as well as the members of *Leptothoe* genus according to our analysis. Herein, we revealed another promising benthic marine cyanobacterium for novel natural products biosynthesis, *Leptothoe*, that warrants further exploration.

## 3. Materials and Methods

### 3.1. Sponge-Associated Strains and Growth Conditions

*Leptothoe kymatousa* TAU-MAC 1615 and *Le. spongobia* TAU-MAC 1115 were previously isolated from the marine sponges *Chondrilla nucula* and *Acanthella acuta*, accordingly, from a rocky sublittoral zone of the North Aegean Sea [21,24]. The strains were purified, and an axenic culture was obtained only for *Le. kymatousa* TAU-MAC 1615 due to difficulties in producing pure cultures stemming from tightly associated heterotrophic bacteria. Axenic and mono-clonal cultures were grown in MN medium [61] for 20–25 days at 20–25 °C, at a photo irradiance of 8–15 μmol photons m^−2^ s^−1^. These strains are maintained in the Microalgae and Cyanobacteria Collection (TAU-MAC) of the Aristotle University of Thessaloniki [62].

### 3.2. Total Genomic DNA Extraction

The genomic DNA of *Leptothoe kymatousa* TAU-MAC 1615 was extracted using a DNA extraction kit (E.Z.N.A.^®^ SP Plant DNA Mini Kit Protocol—Fresh/Frozen Samples, Omega Bio-Tek, Norcross, GA, USA). We harvested 50 mL cultures by centrifugation at 7000× *g* for 10 min, and the pellets were transferred to microcentrifuge tubes. A total of 200 μL of glass beads (two different sizes: 425 to 600 μm and 710 to 1180 μm; Sigma-Aldrich, St. Louis, MO, USA) and SP buffer were added, and the cells were disrupted mechanically with a FastPrep-24 homogenizer (MP Biomedicals, Irvine, CA, USA) at 6.5 m/s for 30 s (2 cycles). The sample of lysed cells was extracted as described in the manufacturers’ protocol.

A total of 50 mL cultures of *Le. spongobia* TAU-MAC 1115 were harvested by centrifugation at 7000× *g* for 10 min, washed twice with washing buffer (50 mM Tris-HCl, 100 mM EDTA, 100 mM NaCl), and transferred to microcentrifuge tubes. After centrifugation (at 7000× *g* for 4 min), the supernatant was discarded, glass beads (two different sizes: 425 to 600 μm and 710 to 1180 μm; Sigma-Aldrich, St. Louis, MO, USA) were added, and the cells were frozen at −80 °C. The sample was thawed at 64 °C and 800 μL of GOS buffer (100 mM TrisHCl (pH 8), 1.5% SDS, 10 mM EDTA, 1% deoxycholate, 1% Igepal-CA630, 5 mM thiourea, 10 mM dithiothreitol) [63] was added. Disruption of the cells was performed using FastPrep at 5 m s^−1^ for 30 s (2 cycles). The rest of the extraction procedure was performed as previously described in detail [63].

The purity, concentration, and quality of the DNA were determined using a Nanodrop ND-1000 Spectrophotometer (Nanodrop Technologies, Wilmington, DE USA), gel electrophoresis, and an Agilent TapeStation (Agilent Technologies, Lexington, MA, USA).

### 3.3. Genome Sequencing and Assembly of Sponge-Associated Cyanobacteria

High-molecular-weight DNA was subjected to library construction (Illumina TruSeq PCR-free 150 bp) and sequenced by the Illumina HiSeq 2500 platform, with a paired-end 100-cycle run (Macrogen Europe, Amsterdam, the Netherlands). The quality of the raw data was initially assessed using FastQC v0.10.1 [64]. Prinseq [65] was used to perform quality filtering, and genome assembly was performed with SPAdes 3.5.0 [66], followed by scaffolding and gap-closing performed with Platanus 1.2.1 [67]. Scaffolds from culture contaminants were identified by Kraken 1.0 [68] and removed using ZEUSS 1.0.2 [69]. Genome assembly statistics were obtained using Assemblathon 2 [70]. Completeness and contamination of the genomes were accessed using CheckM v1.1.3 [71].

### 3.4. Phylogenomic and Phylogenetic Analysis

An alignment of 120 bacterial single-copy conserved marker genes was generated with the Genome Taxonomy Database GTDB-Tk [72] from 90 cyanobacterial genomes, including the two newly sequenced sponge-associated *Leptothoe* genomes as well as 35 genomes registered in GenBank as *Leptolyngbya* or Leptolyngbyaceae and representative taxa of Nostocales, Oscillatoriales, and Chroococcales. A maximum-likelihood phylogenomic tree was constructed by RAxML [73] that was based on the nucleotide substitution model LG +I +G assigned by a BIC calculation in ProtTest [74], with 1000 rapid bootstrap searches.

For 16S rRNA phylogenetic analysis, a dataset consisting of gene sequences belonging to *Leptothoe* genus (>94.5% sequence similarity via BLASTn searches) along with sequences of closely affiliated genera (such as *Salileptolyngbya*, *Nodosilinea*, *Halomicronema*), as well as sequences of other filamentous cyanobacteria was generated. Multiple sequence alignment was performed in MEGA v. 7.0 [75] using ClustalW [76]. The phylogenetic tree was constructed using maximum likelihood (ML) and Bayesian inference (BI). Two 16S rRNA gene sequences of the cyanobacterium *Gloeobacter violaceus* were used as outgroups (GenBank acc. no. AF132790, AF132791). The GTR+I+G model was determined by a BIC calculation in jModelTest 0.1.1 [77] as the most appropriate. The ML analysis was performed in MEGA v. 7 [75]. Bootstrap resampling was performed on 1000 replicates. Bayesian analysis was conducted using MrBayes 3.2.6 [78]. Four Metropolis-coupled MCMC chains (three heated chains and one cold) were run for 10,000,000 generations, the first 2,500,000 generations were discarded as burn-in, and the following datasets were sampled every 1000th generation. Phylogenomic and phylogenetic tree were visualized using the FigTree (V1.4.3) software (http://tree.bio.ed.ac.uk/software/figtree/, accessed on 12 March 2021).

### 3.5. Annotation and Comparative Analyses of Genomes

Open reading frames (ORFs) prediction and annotation were performed using the draft genomes of the two sponge-associated *Leptothoe* strains in the RAST (Rapid Annotation using Subsystem Technology) prokaryotic genome annotation server (version 2.0) [28] with standard procedures. For comparative analysis, five genomes of strains belonging to genus *Leptothoe* were identified in NCBI’s GenBank [79] and selected. Prior to the comparative genomic analyses, all genome datasets were re-annotated using RAST [28] and PROKKA [80]. Subsystems annotation of all seven genomes was performed with the RAST server [28] and SEED tool [29]. CDSs (predicted using RAST) of all seven genomes were subjected to annotation on the basis of clusters of orthologous groups (COGs) of proteins using the on-line server WebMGA (e-value = 0.001) [81]. Pseudogenes were calculated using NCBI’s annotation pipeline. The different classes of mobile elements were analyzed separately. PHASTER [82] was used for phage detection, TransposonPSI (http://transposonpsi.sourceforge.net/, accessed on 12 March 2021) for transposon identification, and ISEScan [83] for identification of insertion sequence elements. In order to detect eukaryotic-like proteins (ELPs) such as ankyrin repeats (ANKs), tetratricopeptide repeat (TPRs), leucine-rich repeats, WD40 proteins, and pyrroloquinoline quinone (PQQ), we searched the annotation files manually using the key words ‘repeats’, ‘Ankyrin’, ‘Tetratricopeptide’, ‘leucine’, and ‘PQQ’ (similar to Karimi et al. [7,34]). Heatmaps for average nucleotide and amino acid identities were estimated using the program GET_HOMOLOGUES [84]. All seven genomes were searched for in terms of the presence of natural product biosynthetic gene clusters (BGCs) using antiSMASH 5.1.1 [85], which was done in order to gain further insight to their metabolic potential.

### 3.6. Data Availability

The *Leptothoe kymatousa* TAU-MAC 1615 Whole Genome project was deposited at DDBJ/ENA/GenBank under the accession number JADOER000000000. The *Leptothoe spongobia* TAU-MAC 1115 Whole Genome Shotgun project was deposited at DDBJ/ENA/GenBank under the accession number JADOES000000000.

## 4. Conclusions

Here, we report the first two draft genomes of sponge-associated filamentous Synechococcales. Our comparative genomic analyses revealed symbiosis signatures of sponge-associated *Leptothoe* such as reduction of their gene content, functional dissimilarities to other host-associated and free-living members of *Leptothoe*, and presence of ELP repeats. Moreover, genome-mining analysis revealed the unique biosynthetic potential of *Leptothoe* with more than 100 natural product BGCs, emerging as an unexplored source of potent marine natural products. Additional studies are needed to identify and characterize these produced compounds. Future research to address the actual functioning of sponge-associated cyanobacteria should include metagenomics, metabolomics, and metatranscriptomics.

## Figures and Tables

**Table 1:** Genome properties and quality metrics of *Leptothoe* strains used for comparative genomic analyses. Subsystem statistics were obtained using the RAST server [28] and SEED tool [29].

Genome Statistics	*Leptothoe kymatousa* TAU-MAC 1615	*Leptothoe spongobia* TAU-MAC 1115	*Leptolyngbya* sp. PCC 7375	*Leptolyngbya* sp. Heron Island J	Leptolyngbyaceae CCMR0081	Leptolyngbyaceae CCMR0082	*Leptolyngbya* sp. SIO3F4
Genome size (bp)	4,068,244	5,242,870	9,422,068	8,064,168	8,660,379	8,890,835	8,111,629
Genome coverage (x)	450	92	30	100	150	136	30
Genome completeness	71.92	70.38	99.73	98.64	99.18	99.46	97.64
Genome contamination	0.28	1.49	1.08	1.49	1.35	1.09	12.31
Scaffolds	18	92	5	119	17	21	1,508
Max scaffold length (bp)	1,235,479	2,951,16	-	-	-	-	-
Min scaffold length (bp)	5043	256	-	-	-	-	-
Mean scaffold size (bp)	226,014	56,988	-	-	-	-	-
Median scaffold size (bp)	161,613	42,147	-	-	-	-	-
Scaffold N50	300,414	99,444	5,859,380	103,122	51,381	147,025	7412
GC (%)	50.5	47.3	47.60	48	47.3	47.4	45.7
Pseudogenes	34	79	-	0	303	260	236
**Mobile elements**	**14**	**68**	**460**	**196**	**115**	**82**	**76**
Phages	0	6	8	4	2	5	3
Transposons	6	32	86	53	36	16	26
Insertion sequences	8	30	366	139	77	61	47
**Subsystem annotation statistics**							
Number of subsystems	185	183	298	285	303	303	300
Number of coding sequences	3638	4790	9182	7785	8186	8350	8779
Coding sequences in subsystems	526 (15%)	616 (13%)	1251 (14%)	1175 (16%)	1204 (15%)	1196 (15%)	1470 (17%)
Coding sequences not in subsystems	3112 (85%)	4174 (87%)	7931 (86%)	6610 (84%)	6982 (85%)	71154 (85%)	7390 (83%)
**Lifestyle**	Sponge-associated	Sponge-associated	Free-living	Free-living	Associated with macroalgae and corals *	Associated with macroalgae and corals *	Associated with macroalgae and other microbes †
**GenBank Accession numbers**	This study	This study	NZ_ALVN00000000.1	NZ_AWNH00000000	NZ_QXHD00000000	NZ_QZCE00000000	JAAHHO000000000

* Isolated from turf samples growing over corals; † isolated from turf samples.

**Table 2:** Eukaryotic-like protein repeats across symbiotic and free-living cyanobacterial genomes.

		Strains	Eukaryotic-Like Domain			
			Tetratricopeptide Repeats	Ankyrin Repeats	Leucine-Rich Repeats	Pyrroloquinoline Quinone
***Leptothoe*** **clade**	*Sponge symbionts*	***Le. kymatousa*** **TAU-MAC 1615**	41	7	6	0
		***Le. spongobia*** **TAU-MAC 1115**	60	3	5	1
	*Host associated*	Leptolyngbyaceae CCMR0081	99	10	10	0
		Leptolyngbyaceae CCMR0082 *Leptolyngbya* sp. SIO3F4	83 93	9 5	9 22	0 0
	*Free-living*	*Leptolyngbya* sp. PCC 7375	102	4	5	0
		*Leptolyngbya* sp. Heron Island J	70	5	8	0
**Other** **cyanobacterial taxa**	**Sponge symbionts**	*Hormoscilla spongeliae GM7CHS1pb*	70	4	56	0
		*Candidatus Synechococcus spongiarum* SH4	3	17	9	0
	**Free-living**	*Nodosilinea nodulosa PCC 7104*	73	1	3	0
		*Leptolyngbya boryana PCC 6306*	70	1	0	1
		*Synechococcus* sp. WH 5701	17	0	0	0
		*Prochlorococcus marinus* CCMP137	6	0	2	0
		*Cyanobium gracile PCC 6307*	12	0	0	0

## Data Availability

The *Leptothoe kymatousa* TAU-MAC 1615 Whole Genome project was deposited at DDBJ/ENA/GenBank under the accession number JADOER000000000. The *Leptothoe spongobia* TAU-MAC 1115 Whole Genome Shotgun project was deposited at DDBJ/ENA/GenBank under the accession number JADOES000000000.

## Supplementary Materials

The following are available online at https://www.mdpi.com/article/10.3390/md19060298/s1, Table S1. Number of genes in subsystems (obtained by RAST server and SEED tool) per *Leptothoe* strain. Table S2. Biosynthetic gene clusters per *Leptothoe* genome (antiSMASH results). Table S3. Compounds produced by *Leptothoe* cyanobacteria extracted from the CyanoMetDB (Jones et al., 2020). Figure S1. Circular view of the genomes of two sponge-associated *Leptothoe* species, generated with CGview (Stothard and Wishart, 2005). Circles from interior to exterior represent GC content and GC skew. Blue circles denote the coding sequences on forward and reverse strands. Figure S2. Average amino acid identity heatmap of *Leptothoe* genomes. Figure S3. Distribution of the categories of functional genes present in seven *Leptothoe* strains on the basis of the cluster of orthologous groups (COGs) of proteins. The alphabetic code for the COG categories is as follows: C, energy production and conversion; D, cell cycle control, cell division, chromosome partitioning; E, amino acid transport and metabolism; F, nucleotide transport and metabolism; G, carbohydrate transport and metabolism; H, coenzyme transport and metabolism; I, lipid transport and metabolism; J, translation, ribosomal structure, and biogenesis; K, transcription; L, replication, recombination, and repair; M, cell wall/membrane/envelope biogenesis; N, cell motility; O, posttranslational modification, protein turnover, chaperones; P, inorganic ion transport and metabolism; Q, secondary metabolite biosynthesis, transport, and catabolism; R, general function prediction only; S, function unknown; T, signal transduction mechanisms; U, intracellular trafficking, secretion, and vesicular transport; V, defense mechanisms.
