# Supplementary material for: Genome Reduction and Secondary Metabolism of the Marine Sponge-Associated Cyanobacterium Leptothoe
Source: Mar Drugs. 2021 May 24;19(6):298. doi: 10.3390/md19060298 (PMC8225149; doi:10.3390/md19060298)
Supplement: Supplementary file 1 [file marinedrugs-19-00298-s001.zip › marinedrugs-1224719-supplementary.pdf]

marine drugs

MDPI

Supporting Information

Genome Reduction and Secondary Metabolism of the Marine Sponge-Associated Cyanobacterium *Leptothoe*

Despoina Konstantinou [1], Rafael V. Popin [2], David P. Fewer [2], Kaarina Sivonen [2], and Spyros Gkelis [1,*]

[1] Department of Botany, School of Biology, Aristotle University of Thessaloniki, Thessaloniki GR-541 24, Greece
[2] Department of Microbiology, University of Helsinki, Viikinkaari 9, FI-00014 Helsinki, Finland
* Correspondence: sgkelis@bio.auth.gr; Tel.: +30-2310-998083

Table of Contents

Table S1. Number of genes in subsystems (obtained by RAST server and SEED tool) per *Leptothoe* strain.

Table S2. Biosynthetic gene clusters per *Leptothoe* genome (antiSMASH results)

Table S3. Compounds produced by *Leptothoe* cyanobacteria extracted from the CyanoMetDB (Jones et al., 2020).

Figure S1. Circular view of the genomes of two sponge-associated *Leptothoe* species, generated with CGview (Stothard and Wishart, 2005). Circles from interior to exterior represent GC content and GC skew. Blue circles denote the coding sequences on forward and reverse strands.

Figure S2. Average amino acid identity heatmap of *Leptothoe* genomes.

Figure S3. Distribution of the categories of functional genes present in seven *Leptothoe* strains based on the Cluster of Orthologous Groups (COGs) of proteins. The alphabetic code for the COG categories is as follows: C, energy production and conversion; D, cell cycle control, cell division, chromosome partitioning; E, amino acid transport and metabolism; F, nucleotide transport and metabolism; G, carbohydrate transport and metabolism; H, coenzyme transport and metabolism; I, lipid transport and metabolism; J, translation, ribosomal structure, and biogenesis; K, transcription; L, replication, recombination, and repair; M, cell wall/membrane/envelope biogenesis; N, cell motility; O, posttranslational modification, protein turnover, chaperones; P, inorganic ion transport and metabolism; Q, secondary metabolite biosynthesis, transport, and catabolism; R, general function prediction only; S, function unknown; T, signal transduction mechanisms; U, intracellular trafficking, secretion, and vesicular transport; V, defense mechanisms.

Table S1. Number of genes in subsystems (obtained by RAST server and SEED tool) per *Leptothoe* strain.

**No. of genes in subsystems**	**TAU-MAC 1615**	**TAU-MAC 1115**	**PCC 7375**	**Heron Island J**	**CCMR0081**	**CCMR0082**	**SIO3F4**
Cofactors, Vitamins, Prosthetic Groups, Pigments	73	68	207	183	199	212	203
Biotin	6	7	12	13	11	11	22
Thiamin biosynthesis	4	5	8	8	8	8	18
Menaquinone and Phylloquinone Biosynthesis	0	8	14	6	14	14	6
Bilin Biosynthesis	8	0	13	9	16	15	12
Cobalamin synthesis	5	0	11	8	11	11	17
Chlorophyll Degradation	1	0	0	1	0	0	1
Heme and Siroheme Biosynthesis	0	8	18	17	18	21	18
Coenzyme B12 biosynthesis	0	0	18	11	15	17	0
Riboflavin, FMN, FAD	7	7	15	15	7	14	19
Pyridoxin (Vitamin B6) Biosynthesis	0	0	7	9	7	8	9
NAD and NADP	11	9	15	15	14	14	19
Folate and pterines	24	15	61	53	55	64	47
Lipoic acid metabolism	2	2	2	2	2	2	2
Coenzyme F420 synthesis	2	0	2	2	2	2	2
Coenzyme A Biosynthesis	3	5	11	14	12	11	11
Cell Wall and Capsule	24	31	32	38	43	47	63
Capsular and extracellular polysaccharides	14	23	11	20	25	25	19
Gram-Negative cell wall components	5	0	0	0	0	0	20
Recycling of Peptidoglycan Amino Acids	4	2	6	5	7	7	4
Murein Hydrolases	0	5	8	5	7	9	10
UDP-N-acetylmuramate from Fructose-6-phosphate Biosynthesis	0	0	0	6	5	5	6
Gram-Positive cell wall components	1	1	2	2	1	1	2
Virulence, Disease and Defense	21	22	44	48	45	41	51
Resistance to antibiotics and toxic compounds	17	12	32	37	32	28	37
Bacteriocins, ribosomally synthesized antibacterial peptides	0	0	2	10	0	0	0
Invasion and intracellular resistance	4	10	10	11	13	13	14
Potassium metabolism	7	11	14	17	16	15	18
Potassium homeostasis	7	11	14	17	16	15	18

Miscellaneous	10	3	17	18	17	17	17
Phages, Prophages, Transposable elements, Plasmids	1	1	8	5	6	8	4
Membrane Transport	33	16	49	63	52	48	58
ABC transporters	0	0	3	6	63	4	5
Protein translocation across cytoplasmic membrane	3	3	2	3	3	3	3
Cation transporters	4	3	8	5	5	5	4
NhaA, NhaD and Sodium-dependent phosphate transporters	0	1	2	2	1	1	2
ECF class transporters	0	0	0	8	0	0	0
Ton and Tol transport systems	10	6	13	19	14	16	2
TRAP transporters	3	3	3	4	4	3	4
Protein and nucleoprotein secretion system, Type IV pilus	13	0	17	16	18	16	21
Iron acquisition and metabolism	1	0	10	8	10	10	8
RNA Metabolism	26	23	52	60	54	49	65
RNA processing and modification	12	5	25	25	26	24	28
Transcription	13	16	23	23	21	20	34
Group II intron-associated genes	1	2	4	12	7	5	3
Nucleosides and Nucleotides	20	25	70	67	70	65	104
Pyrimidines	0	0	13	15	16	15	37
Purines	11	12	38	39	35	34	43
Ribonucleotide reduction	2	2	3	3	3	3	6
Hydantoin metabolism	4	4	9	3	10	7	9
Detoxification	3	7	7	7	6	6	9
Protein Metabolism	73	80	188	178	176	179	188
Protein folding	9	8	12	12	10	10	15
Selenoproteins	2	2	2	5	2	2	6
Protein biosynthesis	23	43	115	107	112	112	116
Protein processing and modification	25	20	27	24	24	26	25
Protein degradation	14	7	32	30	28	29	26
Cell Division and Cell Cycle	0	4	33	26	30	30	54
Motility and Chemotaxis	0	5	0	0	0	0	0
Bacterial Chemotaxis	0	5	0	0	0	0	0

Regulation and Cell signaling	23	34	49	43	50	46	44
cAMP signaling in bacteria	16	22	36	25	40	35	34
LysR-family proteins in Salmonella enterica Typhimurium	0	0	0	1	0	1	0
LysR-family proteins in Escherichia coli	0	0	0	1	0	1	0
DNA-binding regulatory proteins, strays	1	1	0	2	1	0	0
Stringent Response, (p)ppGpp metabolism	2	2	2	2	2	2	2
Programmed Cell Death and Toxin-antitoxin Systems	4	9	11	12	7	7	
DNA Metabolism	32	59	127	85	97	96	106
DNA repair	15	24	62	51	53	53	81
DNA replication	4	2	4	4	4	4	5
DNA recombination	0	0	0	0	0	0	0
CRISPs	0	7	24	11	19	13	7
DNA uptake, competence	2	2	2	2	3	2	2
Type I Restriction-Modification	4	10	12	4	5	9	2
Restriction-Modification System	4	10	12	4	5	9	2
DNA ligases	1	0	1	1	0	0	1
DNA phosphorothioation	0	0	3	2	2	2	0
DNA structural proteins, bacterial	2	4	7	6	6	4	6
Fatty Acids, Lipids, and Isoprenoids	12	1	54	32	54	59	61
Triacylglycerols	2	0	4	2	4	4	0
Phospholipids	0	0	20	0	18	20	0
Fatty acids	0	0	13	13	15	3	20
Isoprenoids	10	1	17	17	17	17	4
Nitrogen Metabolism	13	16	23	21	23	23	25
Cyanate hydrolysis	5	5	6	6	6	6	6
Ammonia assimilation	8	11	17	15	17	17	19
Respiration	29	36	113	75	82	78	59
Biotin	6	7	15	15	14	14	22
ATP synthases	9	9	13	12	10	10	0
Electron accepting reactions	3	3	50	14	21	16	18
Electron donating reactions	5	8	8	15	15	16	12
Cytochrome B6-F complex	0	5	8	9	8	8	6

Soluble cytochromes and functionally related electron carriers	10	11	18	19	19	20	18
Quinone oxidoreductase family	0	0	0	0	1	0	0
Biogenesis of cytochrome c oxidases	0	0	0	1	2	3	2
Biogenesis of c-type cytochromes	2	0	3	3	3	2	3
Stress Response	22	61	84	80	90	90	115
Oxidative stress	17	33	53	51	57	56	73
Osmotic stress	0	1	0	0	0	0	5
Detoxification	5	9	9	9	9	9	12
SigmaB stress responce regulation		7	10	12	10	10	11
Bacterial hemoglobins		15	15	11	15	16	22
Commensurate regulon activation	0	0	0	0	1	1	0
Periplasmic Stress	3	3	15	4	4	4	1
Amino Acids and Derivatives	117	112	257	247	259	265	328
Glutamine, Glutamate, Aspartate and Asparagine Biosynthesis	8	12	23	21	24	23	24
Glutamate dehydrogenases	1	0	1	1	1	1	2
Glutamine synthetases	2	2	3	1	3	3	6
Histidine Biosynthesis	8	0	12	12	12	3	12
Arginine; urea cycle, polyamines	37	10	43	38	45	13	65
S-methylmethionine	0	0	1	1	1	1	1
Methionine Biosynthesis	0	0	22	23	22	23	23
Threonine and Homoserine Biosynthesis	7	9	12	11	12	12	12
Methionine Salvage		3	0	0	0	0	0
Threonine degradation		1	3	2	1	2	1
Lysine Biosynthesis DAP Pathway	0	0	9	9	8	9	11
Cysteine Biosynthesis	7	6	12	13	12	12	12
Creatine and Creatinine Degradation	4	5	7	5	9	8	9
Branched-Chain Amino Acid Biosynthesis	0	0	16	15	16	17	16
Leucine Biosynthesis	4	5	8	7	8	9	9
Aromatic amino acids and derivatives	26	34	35	40	36	37	43
Proline and 4-hydroxyproline	0	0	7	7	8	7	11
Alanine, serine, and glycine Biosynthesis	13	23	34	32	33	37	52
Sulfur Metabolism	0	7	18	19	19	21	20
Phosphorus Metabolism	19	20	33	40	40	38	53

High affinity phosphate transporter and control of PHO regulon	3	3	6	12	9	9	8
Phosphate metabolism	12	13	22	23	26	24	36
Polyphosphate	4	4	5	5	5	5	9
Carbohydrates	71	73	197	177	184	181	241
Central carbohydrate metabolism	10	12	99	80	91	86	124
Aminosugars	2	2	3	3	3	3	3
Di- and oligosaccharides	5	9	14	11	12	14	10
One-carbon Metabolism	4	4	5	5	5	5	4
Organic acids	3	0	0	0	0	0	0
Fermentation	2	5	23	18	21	19	28
CO_2 fixation	29	20	27	32	25	28	44
Sugar alcohols	3	3	5	6	6	5	6
Polysaccharides	6	10	11	11	11	11	11
Monosaccharides	7	8	10	11	10	10	10

Table S2. Biosynthetic gene clusters per *Leptothoe* genome (antiSMASH results)

Strain	Number of genes												
	NRPS	NRPS-like	T1PKS	T3PKS	PKS-like	Bacteriocin	Lassopeptide	Terpene	Cyanobactin	Arylpolyene	Lanthipeptide	LAP	**Total**
Le. spongobia TAU-MAC 1615	0	1	0	0	0	0	0	1	0	1	0	0	**3**
Le. kymatousa TAU-MAC 1115	1	1	2	0	0	3	2	1	0	0	1	0	**11**
Leptolyngbyaceae cyanobacterium CCMR0081	9	3	7	1	1	4	2	2	2	1	0	1	**33**
Leptolyngbyaceae cyanobacterium CCMR0082	5	2	5	2	2	5	2	2	2	1	0	1	**29**
Leptolyngbya sp. SIO3F4	3	2	3	1	0	2	0	3	0	1	0	0	**15**
Leptolyngbya sp. PCC 7375	2	3	0	1	0	2	1	2	1	1	0	0	**13**
Leptolyngbya sp. Heron Island J	4	3	3	0	0	1	2	3	0	1	0	0	**17**
**Total**	**24**	**15**	**20**	**5**	**3**	**17**	**9**	**14**	**5**	**6**	**1**	**2**	

Table S3. Compounds produced by *Leptothoe* cyanobacteria extracted from the CyanoMetDB (Jones et al., 2020).

Compound name	Class of compound	Strain	Reference	DOI
Grassypeptolide E	other cyclic depsipeptide	*Leptolyngbya* sp. RSO3	Thornburg et al. 2011	https://doi.org/10.1021/np200270d
Grassypeptolide D	other cyclic depsipeptide			
Ibu-epidemethoxylyngbyastatin 3	other cyclic depsipeptide			
Dolastatin 12	other cyclic depsipeptide			
Hierridin B	other linear non-peptide	*Phormidium ectocarpi* SAG 60.90	Papendorf et al. 1998	https://doi.org/10.1016/S0031-9422(98)00440-3
Phycoerythrobilin	other linear non-peptide	*Phormidium persicinum*	Chapman et al. 1967	https://doi.org/10.1021/ja00999a058

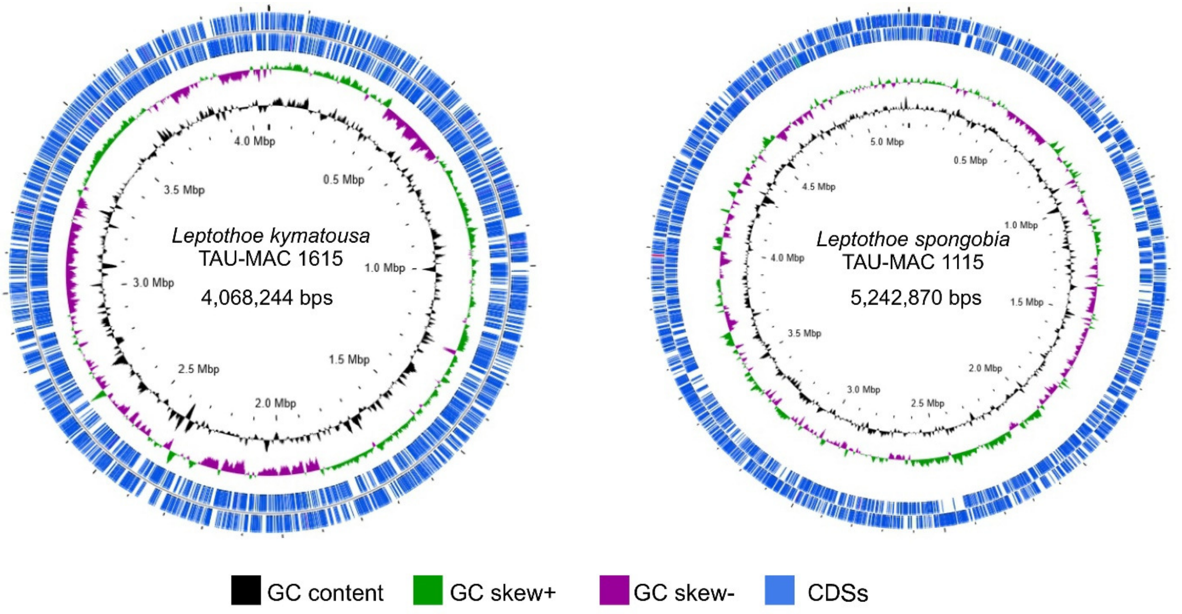

Figure S1. Circular view of the genomes of two sponge-associated *Leptothoe* species, generated with CGview (Stothard and Wishart, 2005). Circles from interior to exterior represent GC content and GC skew. Blue circles denote the coding sequences on forward and reverse strands.

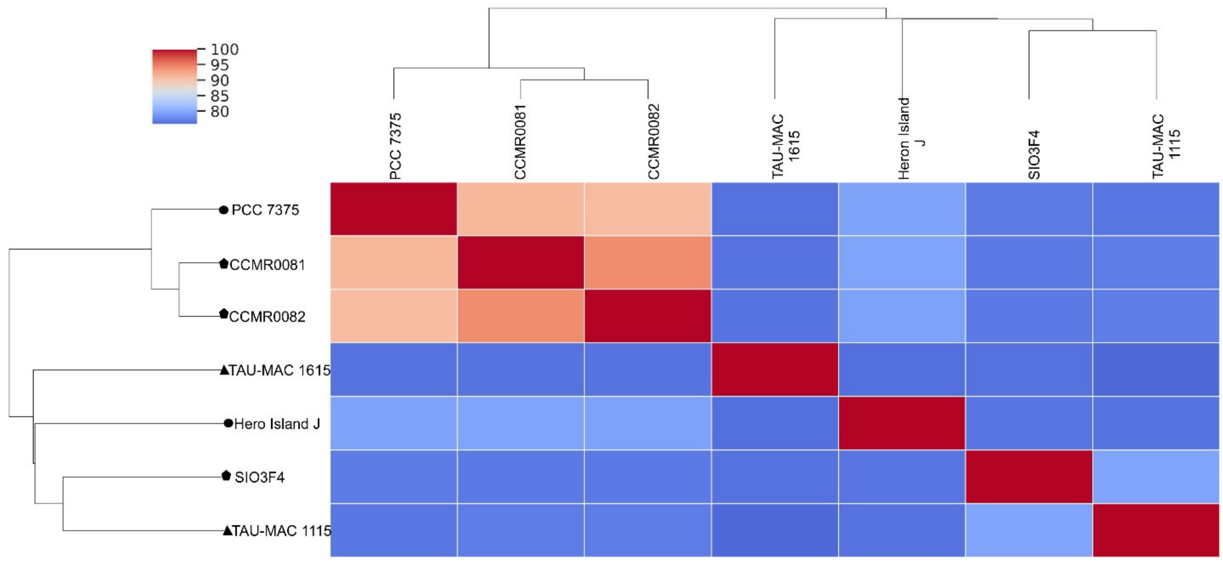

Figure S2. Average amino acid identity heatmap of *Leptothoe* genomes.

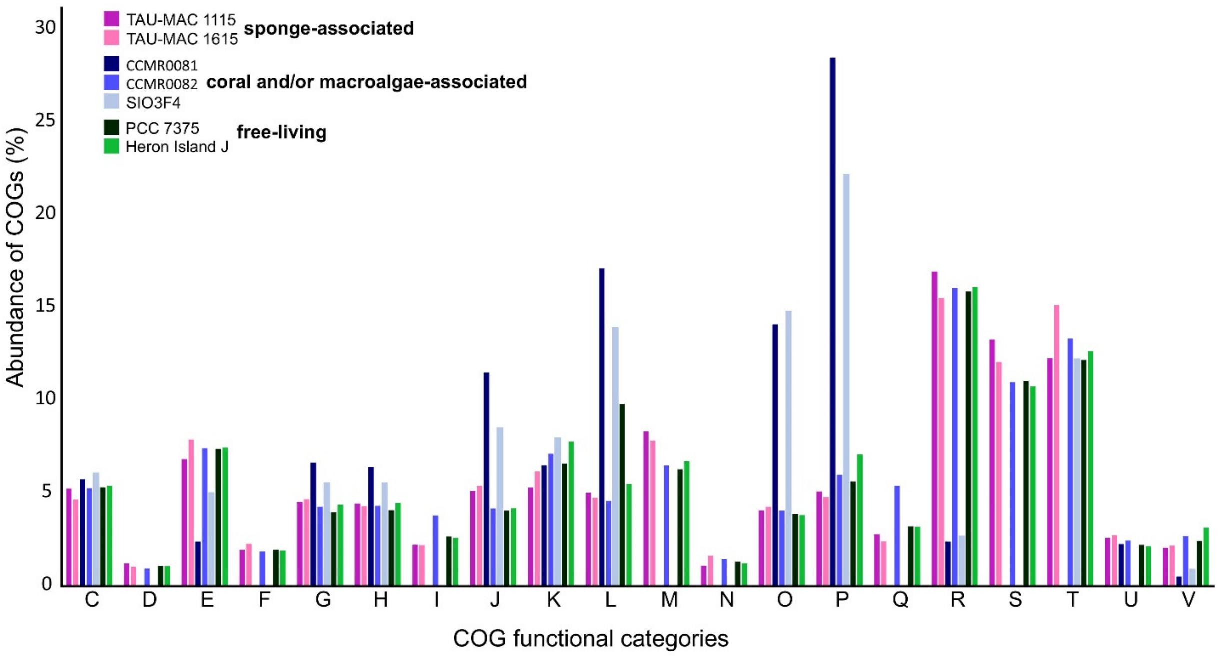

Figure S3. Distribution of the categories of functional genes present in seven *Leptothoe* strains based on the Cluster of Orthologous Groups (COGs) of proteins. The alphabetic code for the COG categories is as follows: C, energy production and conversion; D, cell cycle control, cell division, chromosome partitioning; E, amino acid transport and metabolism; F, nucleotide transport and metabolism; G, carbohydrate transport and metabolism; H, coenzyme transport and metabolism; I, lipid transport and metabolism; J, translation, ribosomal structure, and biogenesis; K, transcription; L, replication, recombination, and repair; M, cell wall/membrane/envelope biogenesis; N, cell motility; O, posttranslational modification, protein turnover, chaperones; P, inorganic ion transport and metabolism; Q, secondary metabolite biosynthesis, transport, and catabolism; R, general function prediction only; S, function unknown; T, signal transduction mechanisms; U, intracellular trafficking, secretion, and vesicular transport; V, defense mechanisms.
